# Permeability of a Macro-Cracked Concrete Effect of Confining Pressure and Modelling

**DOI:** 10.3390/ma14040862

**Published:** 2021-02-11

**Authors:** Wei Chen, Yixuan Han, Franck Agostini, Frederic Skoczylas, Didier Corbeel

**Affiliations:** 1 School of Civil Engineering, Architecture and Environment, Hubei University of Technology, Wuhan 430068, China; chenwei@hbut.edu.cn; 2 CNRS, Centrale Lille, UMR9013—LaMcube—Laboratoire de mécanique multiphysique et multiéchelle, Université de Lille, F-59000 Lille, France; hyx96@jsti.com (Y.H.); franck.agostini@centralelille.fr (F.A.); didier.corbeel@centralelille.fr (D.C.); 3 The State Key Laboratory on Safety and Health of In-service Long-span Bridge, Nanjing 211112, China

**Keywords:** concrete, macro-crack, gas permeability, modelling

## Abstract

The effects of confining pressure are investigated for two samples of a macro-cracked concrete. Samples are first macro-cracked with a splitting tensile strength test (Brazilian) technique. Gas permeability is continually measured under increasing (or decreasing) confining pressure, whereas crack closure (or opening) is recorded with an LVDT (Linear Variable Differential Transformer) device. Despite a mechanical closure of the macro-crack, identified at around 20 MPa confining pressure, gas permeability continues to decrease as confinement is increased. This means that a model of the macro-crack by two parallel planes (using Poiseuille law) cannot be used to represent permeability variations during closure (or opening) of cracks. As a consequence, a physical model is designed in order to simulate with a better consistency the real behaviour of the macro-crack. This simple modelling allows both behaviours, mechanical and hydraulic, under confining pressure, to be simulated with the same set of parameters.

## 1. Introduction

France has 58 nuclear reactors, which supply it with around 75% of its electricity. These reactors are now approximately thirty years old, and EDF (‘Electricité de France and operator of the nuclear reactors) is considering extending their life by several tens of years. EDF is required to carry out ten-year dry air pressurization tests in order to determine the corresponding leakage rates [1,2,3]. The analysis of these tests, together with the identification of the flow mechanisms in a porous matrix or in a cracked (or pre-cracked) zone must make it possible to show that, under accidental conditions, the global leakage will not exceed a certain upper safety limit. The most recent tests have shown that some second-generation containment envelopes (double containment concrete structure) require reinforcement in order to improve their gas tightness. Various reinforcement scenarios are currently being studied; one of them would involve placing an external pre-stressed structure on the outer side of the inner envelope, in order to recompress it—thereby closing the cracks thought to be the cause of the most significant leaks. This kind of issue has been often studied for fragile rocks such as granite [4], soft rocks like argillite [5] and concrete [6,7] for which many experimental studies indicate that the occurrence of micro- or macro-cracks may induce increase in permeability by several orders of magnitude compared to that of the intact material. Such a solution may a priori appear to be attractive. However, pre-stressing a complex structure does not guarantee homogeneous closure of all existing cracks (and could in some case aggravate their condition). On another hand, the compression stress on a macro-crack (which could also be a concrete pre-stressed cladding interface, for example) may restore a sufficiently low permeability.

The present experimental study takes a close look at a macro-cracked concrete, when it is subjected to a compressive confinement (hydrostatic) stress. The experimental equipment developed in our laboratory allowed a macro-crack to be created by splitting, and the gas permeability to be continuously monitored, as a function of the applied stress and/or crack closure [5,8]. These techniques were applied on two cracked samples. They led to identify both hydraulic and mechanical behaviours of macro-cracks submitted to normal stress. A neutral gas, argon, was chosen to evaluate the changes in permeability as the in situ problem relates to air leakage through concrete walls. This fluid is more appropriate than water to precisely indicate the effects of stresses on transport properties [9,10]. This is mainly due to physico-chemical effects that are present with water flow through concrete (or other low porosity rocks) leading sometimes to water permeability being two or three orders of magnitude less than with gas [9]. This phenomenon can often mask the growing or closure of cracks.

The coupled mechanical–hydraulic behaviour of a macro-crack shows a progressive stiffening as well as a sharp permeability reduction with the increase in confining pressure. Both phenomena are clearly irreversible after unloading. It is now well known and demonstrated that a single Poiseuille law is not able to reproduce the fracture hydraulic behaviour as the roughness and the presence of debris play a crucial role in the fluid flow and in the mechanical behaviour.

A simple modelling has therefore been designed to take into account these effects and is presented in this work. The main objective of this modelling is to reproduce both the hydraulic and mechanical behaviours of a macro-crack. These two behaviours have been identified by the gas permeability experiments conducted under loading or unloading. This particular problem has also been often discussed and studied on a hydraulic point of view [11,12,13] but simple and efficient modelling on both aspects, i.e., mechanical and flows are in fact very few (or quite sophisticated [14]) or based on phenomenological relations such as exponential links between stresses and permeability [15].

## 2. Description and Design of the Experiments

### 2.1. Materials

The material used was a concrete with a W/C value of approximately 0.53, with aggregates not larger than 12.5 mm, allowing suitable adaptation to the experimental equipment, and allowing the testing time to be optimised. The composition of the concrete and some properties are given in Table 1. As the tests were designed to evaluate the behaviour of a macro-crack, the type of cement (CEM I 52.5 PM ES, Vicat, Créchy, France) [16] did not have a strong influence on the present study. The concrete had been moulded in a 10 × 10 × 50 cm^3^ formwork and kept in lime saturated water at 25 °C for 6 months, before coring cylindrical samples.

### 2.2. Mechanical Measurements on Fractured Sample

#### 2.2.1. Splitting and Diametrical Fracturing of the Material

**Figure 1:**
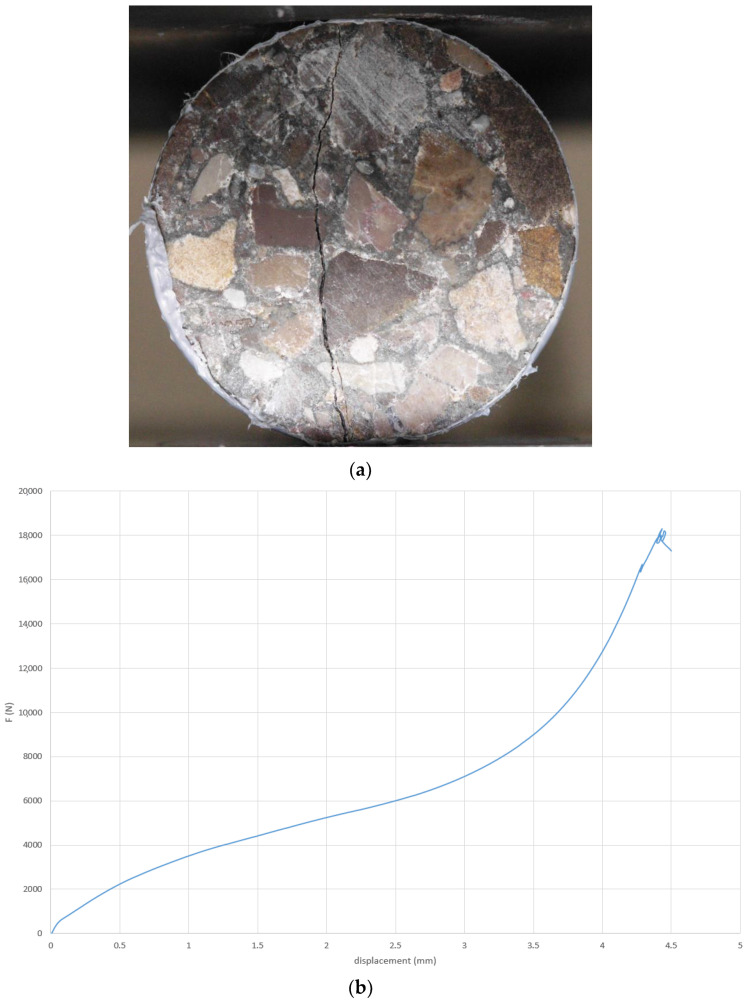
Crack obtained by splitting (**a**) and F-d curve obtained during splitting (**b**).

**Figure 2:**
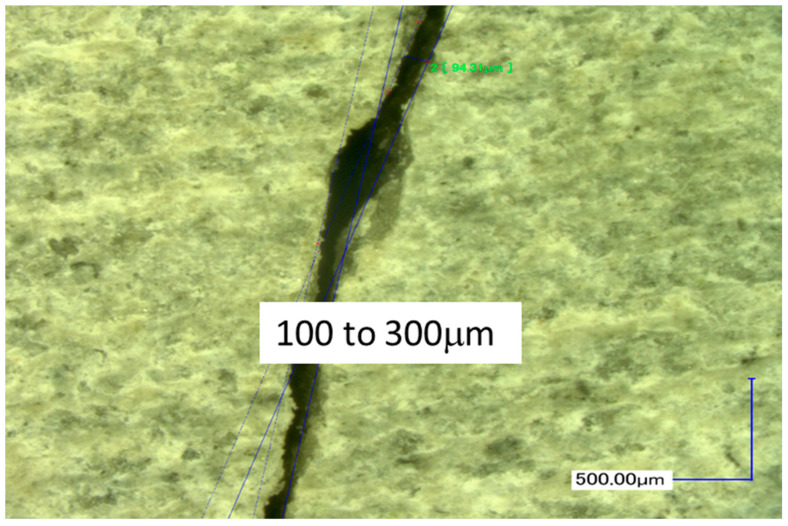
Evaluation of a crack aperture with a Keyence microscope.

To initiate a tensile crack in the test specimen’s axial plane, the Brazilian test is the most appropriate method. Although the crack produced using this method indeed lies in the desired plane (Figure 1a), it was not tried to accurately control its initial width. This then naturally led to disparities in their initial closure, as a function of confining pressure. The tensile strength (see Table 1) was evaluated thanks to the curve F-d in Figure 1b. It was also possible to evaluate the mean initial visible aperture with the use of a Keyence numeric microscope (Figure 2). Two concrete samples were fractured leading to a large crack width. The aperture was in the range 100–300 μm. However, it must be underlined that this crack opening is not homogenous.

It can be noticed that the F-d curve (Figure 1b) is quite different from the usual ones. It is because flexible strips (instead of plywood strips) had been used between sample and press platens in order to minimize lateral sample surface crushing. As a consequence, the measured displacement ‘d’ includes the flexible strip deformation.

#### 2.2.2. Measurement of Crack Closure under Confinement

**Figure 3:**
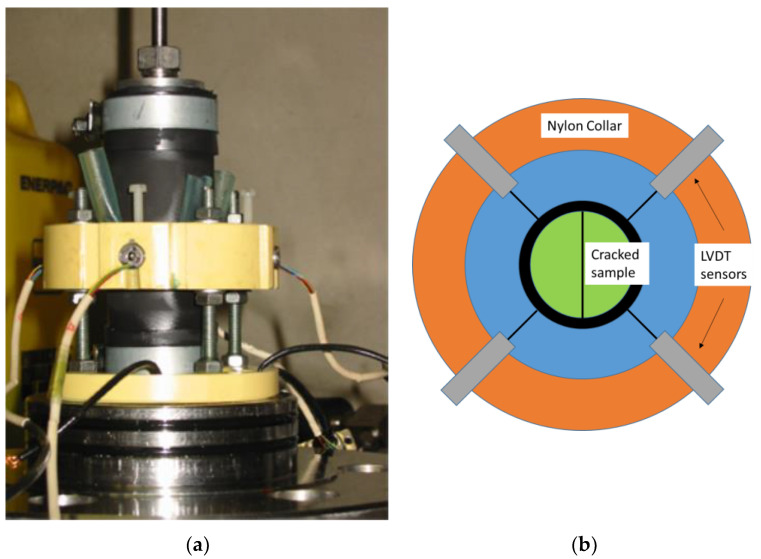
Picture of the device (**a**) Linear Variable Differential Transformer (LVDT) setup, jacket, nylon collars and scheme (**b**).

Here, the aim is to evaluate, in broad terms, the change in crack closure produced by confining pressure, when the specimen is placed in a hydrostatic cell. We measured the mean closure with diametrically opposite LVDT (Linear Variable Differential Transformer, Omega, Manchester, UK) sensors. A picture and a scheme of the setup, which is mounted in the cell, can be seen in Figure 3. This device has already given full satisfaction during trials with other types of material like argillite [5]. However, the test phase begins after calibration. This calibration is crucial since under varying confining pressure, the LVDT device, the jacket and the sample deform. It is thus necessary to take into account these deformations with preliminary tests on (similar) concrete samples and sometimes on steel samples. As a consequence, the results presented in the following report are the actual and sole crack closure and do not include the material strain due to the confining pressure.

Moreover, as mentioned before, the crack opening after the sample splitting is very large, which makes it quite easy to partially close the fracture “by hand”. The sample is enveloped in a thick EPDM (ethylene propylene diene monomer, Borflex, Lille, France) membrane prior to being tested, hence this operation leads to an initial crack closure. Once mounted in the cell, a small confining pressure is applied on the sample (3 MPa in the present case). This pressure, necessary for sealing purposes, is also inducing a partial closure of the crack, which cannot be recorded as it is a setting up phase for the LVDT system. This means that the actual crack initial opening of the sample inside the cell cannot be used in the test analysis.

### 2.3. Measurement of Gas Permeability during Crack Closure (or Opening)

The test involves determining the change in gas permeability under the influence of the confinement inducing crack closure. It was carried out under steady flow rate injection, which is recommended for quite large permeability, as in the case of a fractured sample [17,18]. This method has the advantage of being simple and providing a direct measurement of permeability using Darcy’s law [19,20,21]. The measurement itself and the test conditions involve subjecting the concrete sample to a pressure P_i_ on one side, and allowing the other side to freely drain at a pressure P_o_. The volumetric flow rate is directly recorded at the downstream side of the sample with a Bronkhorst flowmeter. Following the measurement of the downstream flow-rate Q_o_, Darcy’s law is used to determine the permeability K:

(1) $$K=\;\mu \frac{Q_{o}}{A}\frac{2{\mathrm{LP}}_{o}}{\left(P_{i}^{2}-P_{o}^{2}\right)}$$

where A is the sample cross section, L its length and μ the gas viscosity (2.2 × 10^−5^ Pa.s for argon at 20 °C).

**Figure 4:**
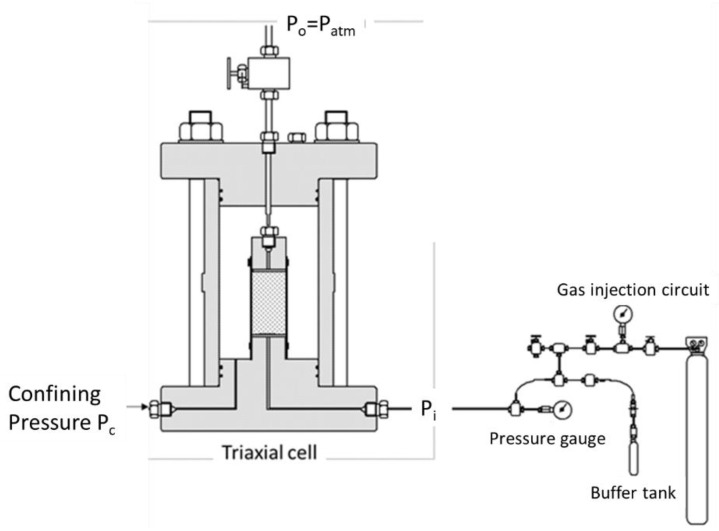
Schematic drawing of the setup used for the permeability measurement.

A sketch of the setup used for the steady state test is shown in Figure 4. It was used to continuously record the permeability during the sample loading.

**Remark** **1.** *In general Darcy’s law is considered to apply if the Reynolds number Re is less than 1. If we consider the highest permeability value obtained during these experiments, we can use a Re calculation with:* $Re=\frac{V\rho \sqrt{K}}{\mu \theta }$ *with V/θ mean flow velocity into porosity (θ porosity here 10.4%), ρ specific mass of argon, μ argon viscosity. For a pressure gradient of 0.5 MPa (and sample height 7 cm), Re is less than 0.001. If the flow is supposed to occur through the crack only, Re is around 0.6, which means that the flow is laminar (Re << 2000). As a consequence, it is assumed that Darcy’s law applies for this kind of experiment.*

## 3. Experimental Results

The experiments were carried out on two different cracked samples. They were cylindrical with 37 mm diameter and 70 mm length. Each of the samples was equipped with an LVDT setup in order to measure the crack’s opening or closure. The maximum level of confining pressure used was around 45 MPa, whereas the gas was injected at a pressure lying in the range between 0.5 and 1.0 MPa. Two main phases can be observed:

(1) A first loading phase which induces a large (and irreversible) crack closure as well as a sharp decrease in gas permeability; and
(2) A second (reproducible) phase, during which the crack’s behaviour is nonlinear elastic, characterised by a bi-unique relationship between confinement pressure and closure, during loading as well as during unloading.

### 3.1. Confinement-Closure of the Crack

**Figure 5:**
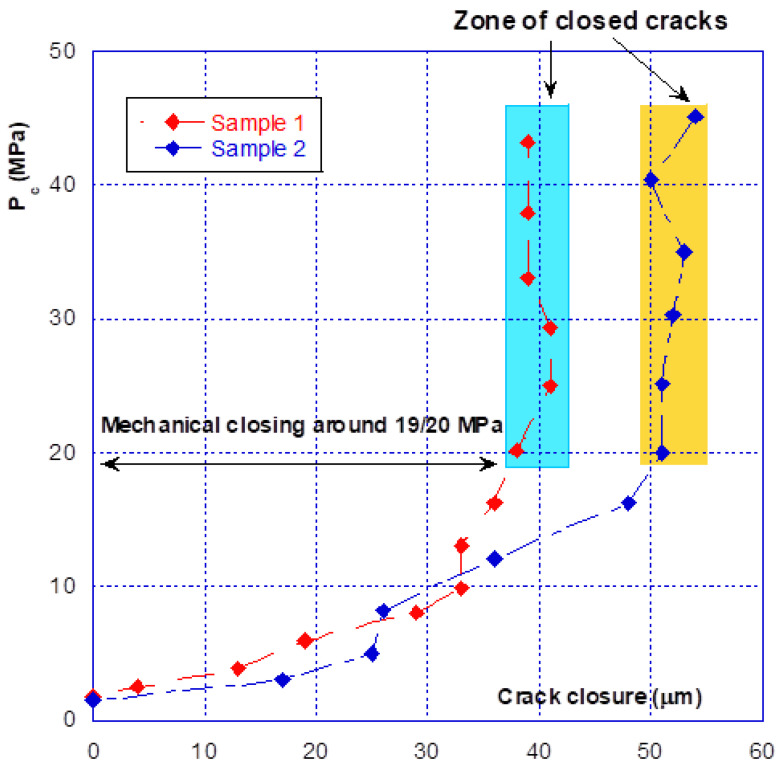
First loading phase for two different samples.

As briefly described above, there is an initial crack closure phase that should be distinguished from the following phases, which are reproducible and involve much smaller amplitudes. Figure 5, corresponding to the first loading phase for the two samples, illustrates this statement. The total crack closure is 40 μm for the sample 1 and 50 μm for sample 2. These values are, as expected, lower than the initial aperture obtained just after the splitting operation (see comments in Section 2.2.2). It can be seen that mechanical closure occurs at a confinement pressure of around 20 MPa, with the closure then varying so little (the curve is nearly vertical) that these small variations and “oscillations” are probably due to the limitations of the sensors themselves. This result clearly shows that between 20 and 45 MPa of confining pressure, there is no more relative movement between the crack edges and that, on a global point of view, the cracks are closed.

Nevertheless, as it will be seen in the following, there are two distinct behaviours for the crack—mechanical (i.e., closure/opening with pressure) and hydraulic (change in permeability). For the first part the (global) mechanical closure occurs around 20 MPa of confining pressure. However, the complete closure of macro-cracks is not possible because there will likely be blockages caused by loose aggregate or debris, for example, or if there have been relative movements causing misalignment between the two sides of the cracks. This is expected to happen in the Brazilian splitting test. Some areas of the crack may be propped open until the material lodged inside is crushed. This incomplete closure of the crack will explain why permeability continues to decrease as confinement increased beyond 20 MPa and why the permeability of the cracked sample remains much higher than the permeability of the matrix.

**Figure 6:**
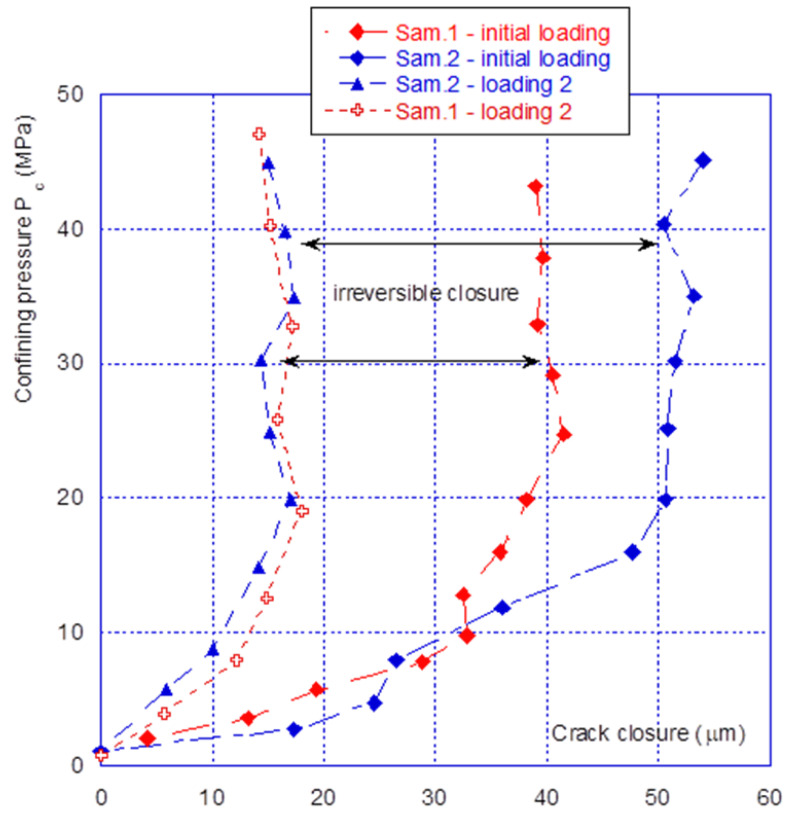
Closure comparison for two samples—initial and reproducible closure.

The opening–closing amplitude after the first confinement is small, at approximately 15 µm. From Figure 5, comparison of the initial closure of the two tested samples shows that they are highly similar, with sample n° 2 having a slightly stronger amplitude. However, from Figure 6 it can be seen that after several seconds of loading–unloading, the initial differences are reduced, and the closure behaviour of the two samples is very similar. It must be pointed out that, at this stage, the crack behaves reversibly. As a preliminary conclusion, it can be stated that after several loading–unloading cycles, the mechanical behaviour of the two different samples is virtually identical, even though the initial closures were different.

Note—as underlined before, the testing device and method do not allow the value of the initial crack opening to be determined (i.e., at the beginning of the loading). The average opening obtained after splitting can be evaluated but is useless with regard to what is actually measured. On another hand, the physical relative displacement (measured with the LVDT) between the crack edges is null between 20 and 45 MPa of pressure. This obviously means that both opposite surfaces of the cracks are in mechanical contact and that no macroscopic kinematical parameter could be used to described the crack closure. It can then be said that from 20 MPa of confining pressure, the cracks are closed. As a consequence, the crack closure, measured from 2 to 20 MPa, can be used at the initial mean crack opening. However, this does not mean that the contact between the crack on opposite surfaces is total and perfect. This particular aspect will be used in the following modelling.

### 3.2. Relationship between Confinement and Gas Permeability

**Figure 7:**
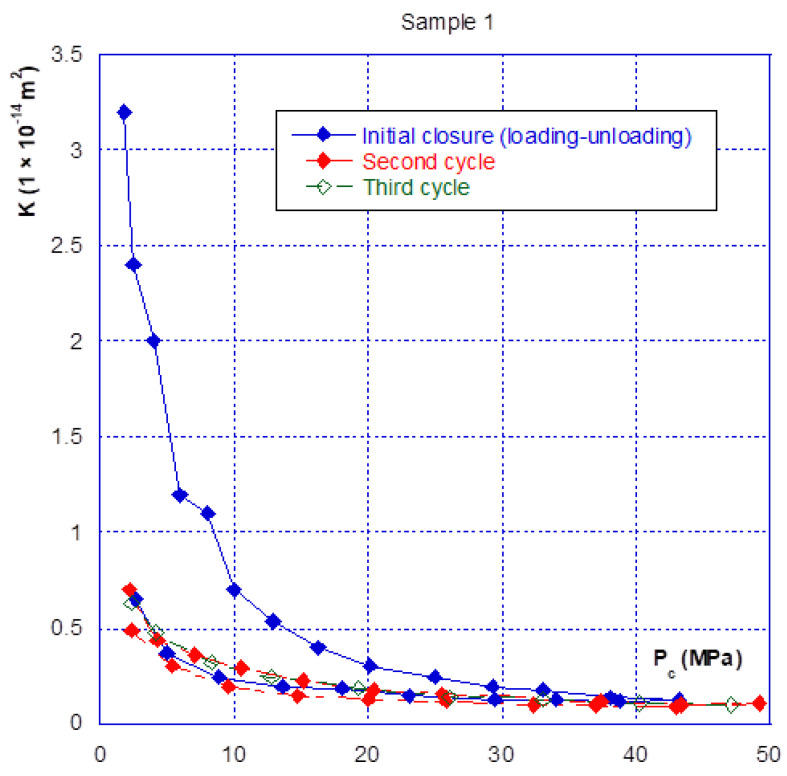
Sample n°1—successive closure cycles.

**Figure 8:**
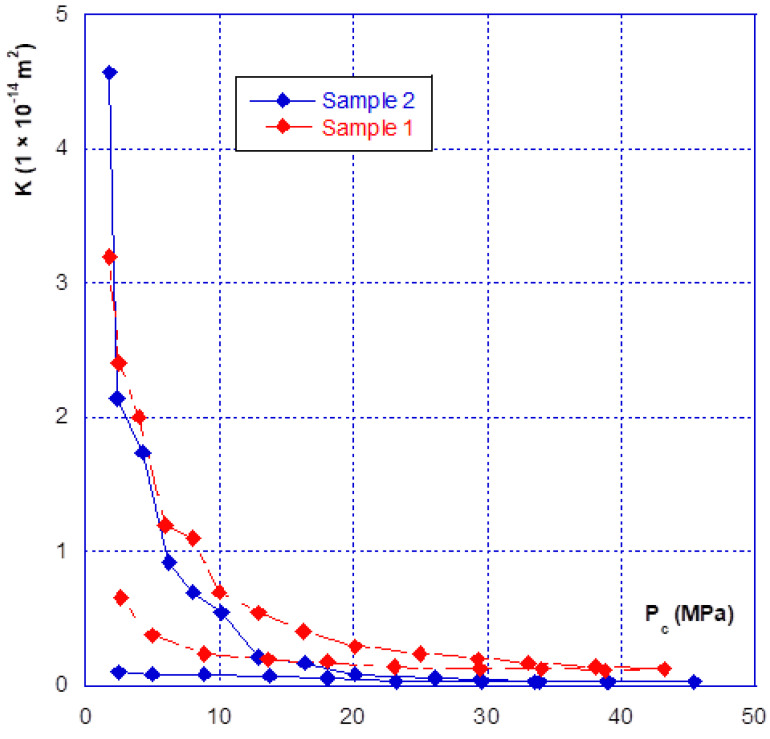
Comparison of the fracture permeability of the two different samples.

As for the mechanical behaviour, there are two phases in the hydraulic behaviour of a fracture under confining pressure—the initial closure phase, and the following phases. Figure 7 clearly illustrates this aspect, showing that during the initial fracture closure cycle, the change in permeability is very sudden, up until the occurrence of mechanical closure (at around 20 MPa), and it is irreversible. This behaviour is confirmed with the results presented in Figure 8 where we compared the permeability of both samples. The sample 2 permeability is slightly higher than that of sample 1 at the beginning of the loading (low confining pressure). This is consistent with the differences in crack closure, which is greater for sample 2 (i.e., its initial opening is thus higher). In the case of the other cycles, whatever the injection pressure, the relationship between permeability and confining pressure is restricted to a very narrow band, and becomes almost reversible. It can thus be noted that the unloading phase of the cycle remains inside this band (Figure 7 and Figure 8). This is consistent with the mechanical crack behaviour that is also reversible after the first phase of (large) closure.

**Figure 9:**
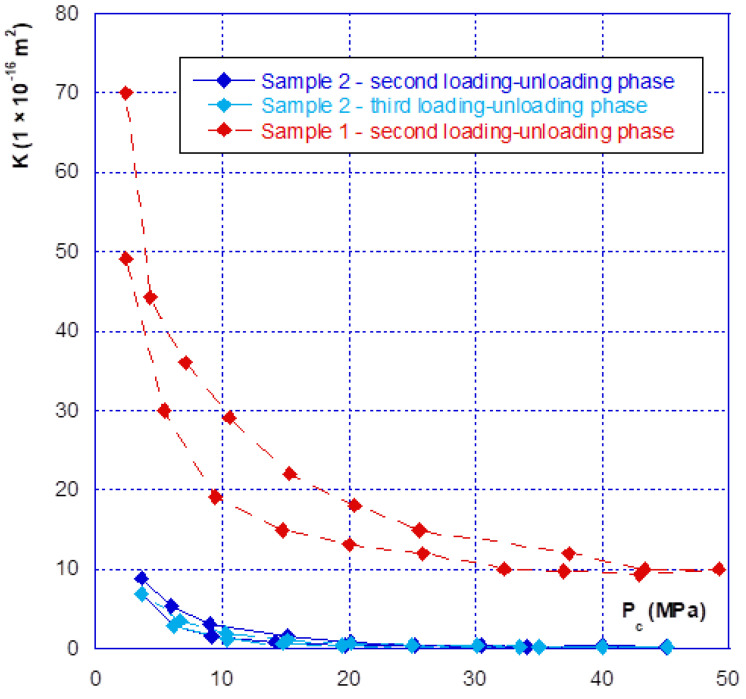
Variations in permeability obtained after the first phase of loading–unloading.

**Figure 10:**
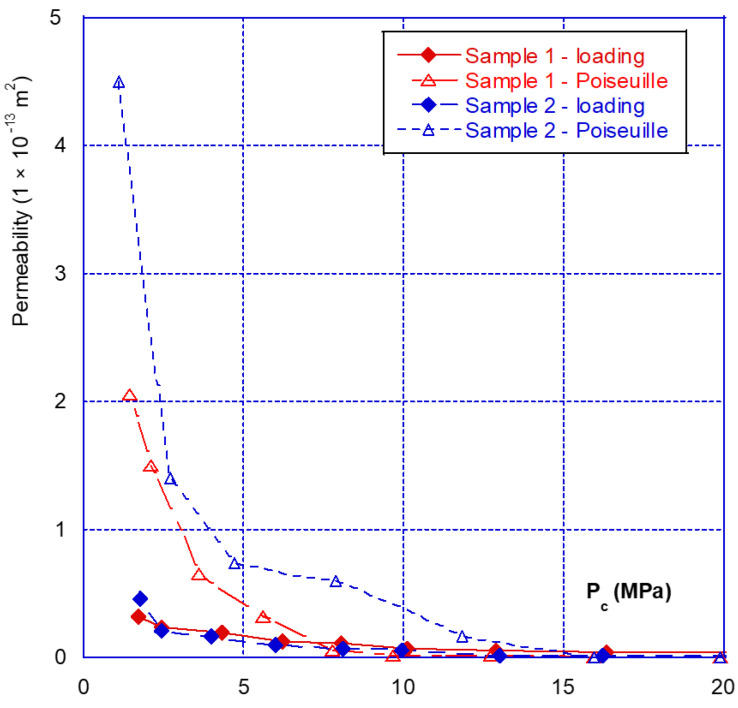
Comparison of measured permeability with the one derived from Poiseuille law—for both samples (loading phases).

Finally, even when the crack is mechanically closed (see note in Section 3.1), the permeability varies only slightly, with a value of approximately 10^−15^ m^2^, and thus remains much greater than the matrix permeability, which is of the order of 10^−17^ to 10^−18^ m^2^. However, when the permeability of the two different samples is compared (Figure 9), a ratio of approximately one order of magnitude is found, even after mechanical closure. This observation leads to several comments:

(1) The Poiseuille law had been applied to two sheets separated by a distance equal to the width of the fracture. The Figure 10 gives the comparison of the real (experimental) results and simulations with Poiseuille law up to 20 MPa. It can be seen that the use of this law, with such a geometry, was not able to simulate the actual crack behaviour as it overestimates the permeability, especially at the beginning of loading. It is why a new modelling of the crack geometry had been designed in order to better estimate its permeability during loading. This modelling is presented in Section 4;
(2) These experiments show with certainty that, for clean fractures such as those described here, there will always be preferential flow channels, which will not be closed by a normal level of stress (45–50 MPa is already quite a high value). At the end, these flow channels will make the difference between both sample permeability, even if the fracture is closed; and
(3) At high levels of stress (greater than that required for mechanical closure—Figure 9), only a few percent of the initial permeability remain, which then varies only slightly, and “slowly”.

In relation to the problem of reinforcing the containment envelopes of nuclear power stations for the purposes of gas tightness, these results unambiguously show that a solution involving the application of a pre-stressed structure outside the dome would, at best, allow only partial restoration of the wall’s gas tightness. Even when mechanical closure is achieved, the effective permeability remains much higher than the matrix permeability of the material itself.

## 4. Crack Behaviour Modelling

### 4.1. Design of the Model

As seen before, three main phases can be observed with regard to the mechanical and hydraulic (permeability) behaviours of a macro-fracture under confining pressure:

- A first closing phase under low confinement during which the gas permeability sharply decreases (Figure 7 and Figure 8);
- A progressive hardening phase until the occurrence of the mechanical closure. This closure is linked to a rapid increase in confining pressure leading to a low amplitude crack closure. During this phase, the gas permeability is slightly decreased but is never null (Figure 7, Figure 8, Figure 9 and Figure 10);
- There is a clear irreversibility during unloading as the crack is partially reopening and the permeability slightly increases while remaining lower than the initial one (Figure 7 and Figure 8).

**Figure 11:**
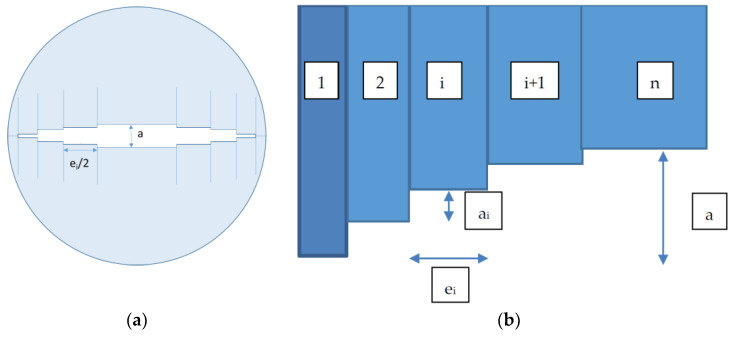
Geometrical modelling of the fracture. (**a**) sample cross section; (**b**) geometrical parameters.

As a consequence, any modelling has to be consistent with these three points. Moreover, it is obvious that the fracture surface cannot be a plane and must include some roughness linked to asperities. If these asperities are of different height, one can suppose that more and more asperities will be in contact, leading to the fracture hardening. The geometrical model is therefore presented in Figure 11.

To simulate an increasing number of asperities getting in contact under the confining pressure rise, the fracture is composed of a succession of n “elements” with a length L_i_ and a width e_i_. The difference L_i−1_ − L_i_ is “a_i_” as indicated in Figure 11. The global initial macro-crack opening is “a”. The sample is cylindrical with a diameter is D and a total length L. The width e_i_ as well as the height difference a_i_ are supposed to vary according simple geometrical rules: e_i + 1_ = p e_i_ and a_i + 1_ = r a_i_. The e_i_ increase allows to accentuation of the hardening when the confining pressure is growing. i.e., more and more elements are in contact. Using these geometrical rules, one can obtain two relations:

$$e_{1}=\left(\frac{p-1}{p^{n}-1}\right)D$$

where e_1_ is the first element width

$$a=\left(\frac{1-r^{n-1}}{1-r}\right)a_{2}$$

Note—as a purpose of the example and in the following, we have chosen p > 1 and r < 1, but any other choice can be made to best fit the real behaviour of a crack.

**Figure 12:**
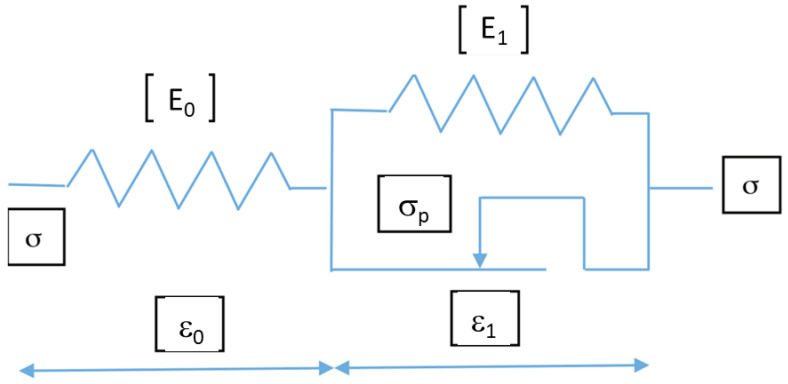
Uniaxial behaviour of an element.

The model also has to take into account the mechanical and hydraulic irreversibility. Both are linked, since a partial re-opening of the crack during the unloading phase (mechanical cause) leads to a lower permeability for the same confining pressure. A simple elasto-plastic behaviour was therefore chosen to simulate these irreversibilities (Figure 12).

The « dry friction part » has a threshold limit σ = σ_p_ (or alternatively ε = ε_p_ = σ_p_/E_0_) leading to the following relations for loading: ε = ε_0_ + ε_1_

If σ < σ_p_ then ε = σ/E_0_ and when σ > σ_p_ then σ = σ_p_ + E_1_ ε_1_ with σ = K ε+ K/E_1_ σ_p_ in which K = E_0_E_1_/(E_0_ + E_1_). The elastic strain is recovered for unloading leading to: ε = −σ/E_0_.

### 4.2. Behaviour of the Crack for Loading (Increasing Confining Pressure)

For this process it was more convenient to control the crack closure δ (δ < a) and to deduce the corresponding pressure P. To simplify the writing, one can suppose that this closure results in “j” elements being in contact, “j” is deduced from Equation (2) below:

(2) $$\frac{\delta }{a}=\left(\frac{1-r^{j-1}}{1-r^{n-1}}\right)$$

The element i (with i < j) axial strain of the element “i” (with i < j) is therefore given by:

(3) $$\varepsilon_{i}=\frac{\Delta_{i}}{D}=\frac{1}{D}\left(\delta -\frac{1-r^{i-1}}{1-r^{n-1}}a\right)$$

where Δ_i_ is the length variation of the element “i”.

It can be observed in Equation (3) that the strain ε_i_ is artificial as the real initial length of the element “i” is not known and surely different from one element to another. To simplify this, the core diameter “D” was chosen as this initial length. From a practical point of view, it will be seen in the following that this choice has no real importance on the simulation because it can be counterbalanced by the values of the moduli E_0_ and E_1_ involved in the calculations. From a given value “δ”, the uniaxial stress in every element can be calculated. It is first necessary to identify the strain state for element “i”, i.e., plastified or not. On the other hand, it is just necessary to verify whether ε_i_ > ε_p_ or not. Practically, one can use the parameter α such as ε_p_ = α a/D. If δ/a > α then « m » elements are plastified and « m » is the maximum value, which verifies the following inequality:

(4) $$r^{m-1}\geq \left(1-\left(1-r^{n-1}\right)\left(\frac{\delta }{a}-\alpha \right)\right)$$

As soon as « m » is known, the stress for every element can be calculated according the value of “i”:

When I ≤ m, σ(i) is calculated from Equation (5):

(5) $$\sigma \left(i\right)={K\varepsilon }_{i}+\alpha \frac{K}{E_{1}}\frac{E_{0}a}{D}$$

And when m < i < j, σ(i) is deduced from Equation (6):

(6) $$\sigma \left(i\right)=E_{0}\varepsilon_{i}$$

where σ(i) is null for the other elements.

These stress values σ(i) are calculated and linked with the confining pressure P by the use of Equation (7):

(7) $$P=\frac{1}{D}\sum_{1}^{j}\sigma \left(i\right)e_{i}$$

There is a reversible re-opening λ_max_ of the crack after complete unloading. This re-opening can be easily deduced from the stress in element 1 for which a complete unloading will lead to λ_max_ = D σ(1)/E_0_.

### 4.3. Behaviour of the Crack for Unloading (Decreasing Confining Pressure)

If a reopening λ < λ_max_ takes place leading to the strain Δε = λ/D in every element, two situations can occur for element number « i »: a reopening such as there is no longer stress in the element or there is still contact and stress. If one supposes that « f » elements are remaining in contact then the elastic part of the strain ε_f_^e^ should verify the inequality: ε_f_^e^> Δε with ε_f_^e^ = σ(f)/E_0_. The residual stress in these « f » elements simply derives from this calculation by:

(8) $$\sigma_{r}\left(i\right)=\sigma \left(i\right)-E_{0}\Delta \varepsilon$$

As in the other elements there is no stress, the confining pressure, linked to the reopening λ is now:

(9) $$P_{r}=\frac{1}{D}\overset{f}{\underset{1}{\sum }}\sigma_{r}\left(i\right)e_{i}$$

### 4.4. Hydraulic Behaviour

In the modelling (Figure 11), each element defines two parallel planes with a width e_i_ and a distance d_i_ between them. It will thus be assumed that this modelling involves a Poiseuille flow type with no interaction between the elements. Within this simple frame it is easy to calculate the flow rate Q_i_ with a pressure gradient ΔP/L:

(10) $$Q_{i}=\frac{1}{12\mu }\frac{\Delta P}{L}e_{i}d_{i}^{3}$$

The total flow rate is composed for all the elements and obtained by:

(11) $$Q=\frac{1}{12\mu }\frac{\Delta P}{L}\sum_{1}^{n}e_{i}d_{i}^{3}$$

As it was seen before, the tests carried out in our experiments are based on the global sample permeability K measurement. This property is then deduced from Equation (12).

(12) $$K=\frac{1}{12A}\sum_{1}^{n}e_{i}d_{i}^{3}$$

A is the sample cross section and μ the fluid viscosity.

One can consider three states to calculate K—initial, during loading and for unloading.

Note—it can be seen that the matrix gas permeability is neglected in the Equation (12). This is a reasonable assumption as the matrix gas permeability is two or three orders of magnitude less than K.

## 5. Initial State

We calculated the distance d_i_ as soon as the geometrical modelling was chosen. In the initial state d_1_ = 0 and for i > 1:

(13) $$d_{i}=\frac{\left(1-r^{i-1}\right)}{\left(1-r\right)}a_{2}$$

the permeability is now obtained with Equations (12) and (13).

## 6. Loading Phase

As seen before, this loading is characterised with the closure “δ” leading to “j” elements being in contact. The distance d_i < j_ is null, i.e., no flow between these “j” elements. The distance between the remaining open elements (for i > j) is (d_i_ – δ) leading to a new calculation for the permeability:

(14) $$K_{c}=\frac{1}{12A}\sum_{j+1}^{n}e_{i}{\left(d_{i}-\delta \right)}^{3}$$

## 7. Unloading Phase

Unloading has been previously characterized with a reopening λ leading to “f” elements that are still in contact, i.e., with no participation to the flow. There are now two situations to be considered—the elements “i” such as i > j were not in contact during the loading, the aperture after unloading is becoming (d_i_ − δ + λ) and the elements “i” such as f < i < j for which the aperture after unloading (and recovering of the elastic strain) is now (λ − Dε_i_^e^). The permeability can be then deduced from Equation (15):

(15) $$K_{d}=\frac{1}{12A}\left(\sum_{j+1}^{n}e_{i}{\left(d_{i}-\delta +\lambda \right)}^{3}+\sum_{f+1}^{j}e_{i}{\left(\lambda -{D\varepsilon }_{i}^{e}\right)}^{3}\right)$$

### 7.1. Simulation of Thereal Fracture Behaviour

The aim of this last part is to evaluate the efficiency of this kind of modelling of the two permeability tests presented in Section 3. The modelling presented above has been used and programmed with the Excel program. The main parameters, used for both samples, are given in Table 2. It can be seen that they are very few and most of them have a clear physical meaning. “a” is the initial crack opening and “δ” the closure that was recorded during the confining test. “p” and “r” are geometrical parameters that allow us to take into account different crack geometry and roughness. E_0_, E_1_ and ε_p_ are mechanical properties. The number of elements “n” is quite small but revealed to be large enough to bring a consistent modelling.

**Figure 13:**
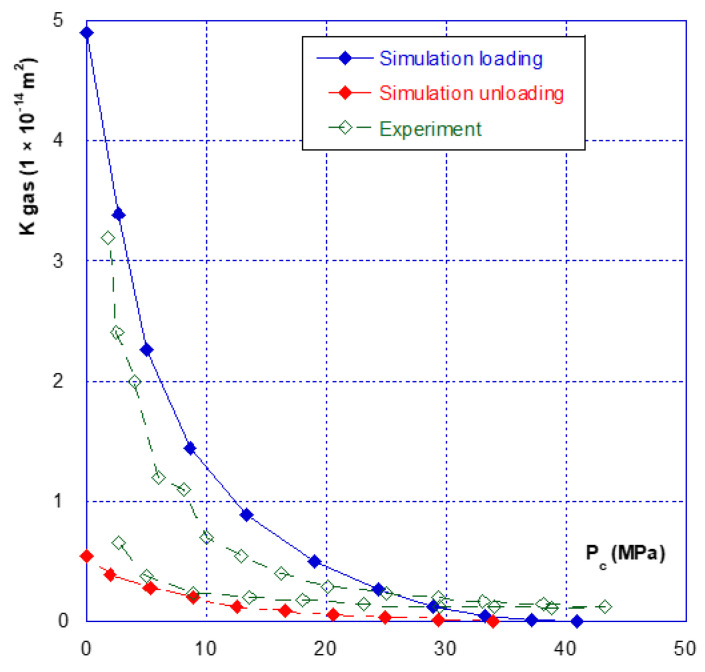
Permeability simulations for sample 1.

**Figure 14:**
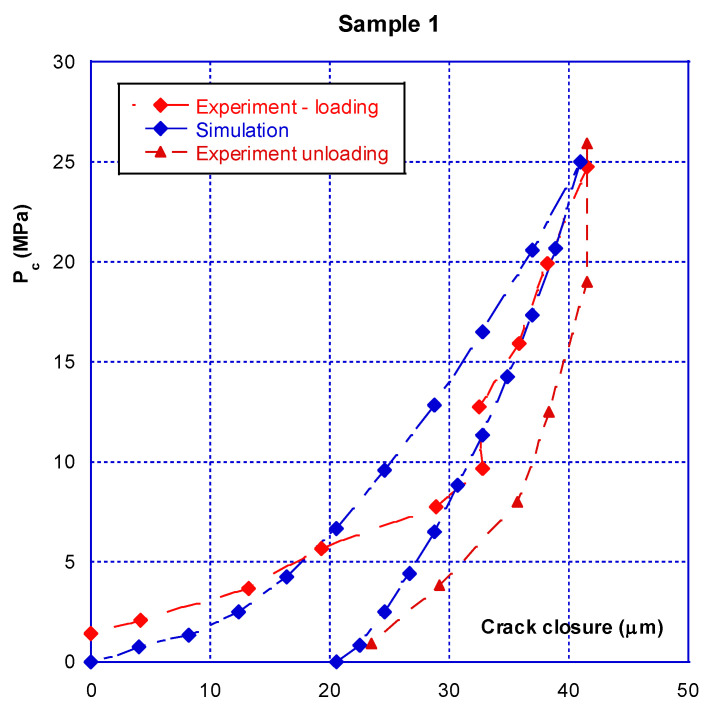
Comparison of real results and simulations for the crack closure of sample 1.

**Figure 15:**
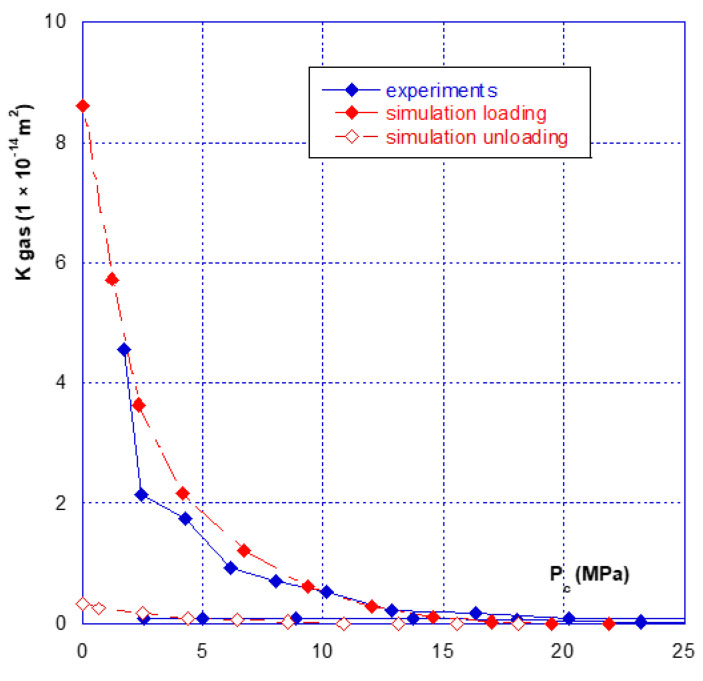
Permeability simulations for sample 2.

**Figure 16:**
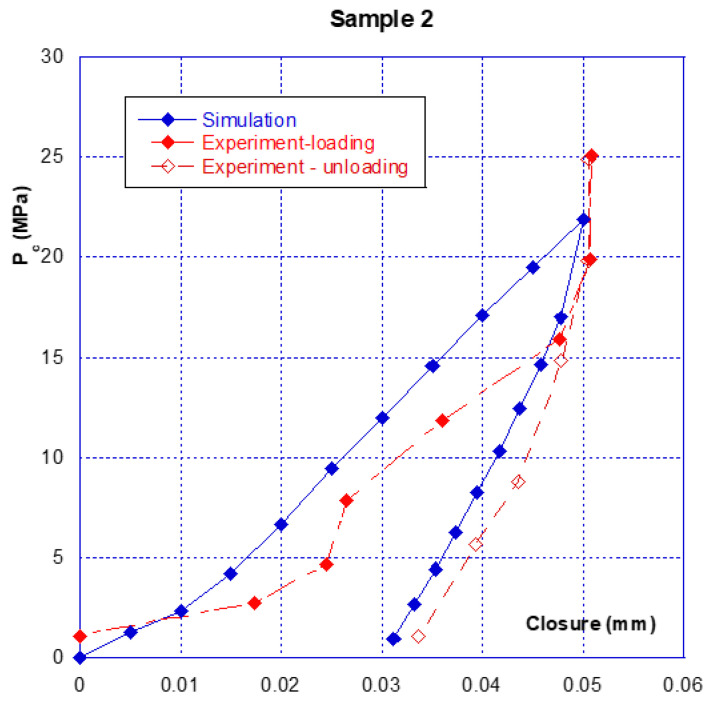
Comparison of relative results and simulations for the crack closure of sample 2.

The calculated results and their comparisons with actual ones are given in Figure 13 and Figure 14 for sample 1 and Figure 15 and Figure 16 for sample 2. It is useful to underline that the parameters given in Table 2 are the same for both simulations, i.e., hydraulic (permeability) and mechanical (crack closure). It can be seen in Figure 13 and Figure 15 that the modelling can (quite easily) reproduce the hydraulic test (for both loading and unloading) and is consistent with the absolute permeability values which were measured during the tests. This is a clue that the physical assumptions, used to design this modelling, are relevant. Compared to a basic use of Poiseuille’s law (Figure 10), it is clear that the present modelling brings a strong improvement. The elasto-plastic behaviour of the elements is also able to describe the irreversibility observed for both mechanical and hydraulic crack behaviour when it is unloaded.

### 7.2. Note about the Modelling and the Choice of Parameters

This simple and first step of modelling is intended to show that the classical Poiseuille law can be deeply improved by the choice of a fracture geometry that can reproduce a large part of its mechanical and hydraulic behaviours, despite its great complexity (i.e., roughness, presence of debris, potential layering, etc.). This modelling is based on some ideal representation with elements that are progressively put into contact to reproduce the stiffening of the fracture under increasing confining pressure and a sharp permeability decrease. It could be enhanced, with the same principle, by the use of different kinds of element distribution rules but it was not the purpose of the present study. The geometrical parameters (a, p and r) were chosen to match the initial permeability. E_0_, E_1_ and ε_p_ were roughly optimized to be close to the real mechanical behaviour of the fracture. One can object that, for example, E_0_ is (a priori) not the real material for Young’s modulus and different for the two samples, which come from the same concrete. On another hand, it must be kept in mind that the element strain, introduced in this modelling, is quite artificial and has to represent multiple phenomena (crushing of particles, possible layering etc.). It is, however, quite logical that E_0_ for sample 2 is less than E_0_ for sample 1 as the global closure of the fracture for sample 2 is higher than the one for sample 1, at the same level of confining pressure.

The mechanical simulations are given in Figure 14 and Figure 16. These calculations were carried out up to 20–25 MPa of confining pressure, i.e., when the cracks reach the mechanical closure. The simulated behaviour of these cracks is also consistent with the measurements, as well under loading or unloading. This shows that such a simple modelling is promising as, even far from being optimized, it is able to reproduce a complex behaviour of a micro-crack on both aspects—hydraulic and mechanical.

## 8. Conclusions

The present study describes the results of several months of tests carried out in our laboratory. Our macro-fracture test methodology was effective, and allowed useful results to be obtained concerning the fracture’s hydraulic behaviour—even when it is mechanically closed, a macro-crack always has a much greater permeability than that of the matrix. On another hand, this means that a macro-cracked concrete will never recover its initial (matrix) gas permeability even if a high level of pressure is applied to close the crack. Following initial loading, the overall opening and closing amplitudes were small, of the order of 20 μm. After initial closure, the fracture’s behaviour became nonlinear elastic and variations in its permeability were very well correlated with the confining pressure. It is difficult to express the permeability as a function of the crack’s width, since this continues to change (very slightly) when the fracture is closed. However, these tests also show that in order to develop an expression for the permeability as a function of a single parameter, such as mechanical damage, it would be necessary to include information related to the crack’s width (or the confining pressure).

The presence of intra-fracture channels, which could explain the non-zero permeability observed following mechanical closure, can be modelled by imperfect contacts between particles producing roughness. A “simple” modelling has thus been developed in order to take into account the progressive stiffening of the crack and the irreversible behaviour observed when it is unloaded. The strategy was used for a succession of elements of different sizes and width and to assume that their axial behaviour is elasto-plastic. To be applied, this model needs very few parameters and the calculations derived from its use have led to satisfactory results when compared to actual experiments. Thanks to the continuous gas permeability recording as a function of crack closure, it had been possible to identify the model parameters. This allowed the simulation of both the hydraulic and mechanical behaviours of a macro-crack with the same set of parameters. On another hand, based on the same principle, more sophisticated tools could be used to enhance such a simple modelling—different distribution of the elements (normal distribution, random distribution, etc.), association in parallel or in series. It was nevertheless not the purpose of the present work.

## Figures and Tables

**Table 1:** Concrete composition and (mean) basic properties.

Constituents	Proportion (kg/m^3^)
Cement: CEM I 52.5 PM ES	350
Sand 0/4	764
Gravel 4/12.5	1075
Water	185
**Property**	**Value**
Young modulus E	39 GPa
Compressive strength	60 MPa
Tensile strength (splitting test)	4.4 MPa
Porosity	10.4%
Gas permeability K (drying at 105 °C)	1 × 10^−18^ m^2^ < K < 1 × 10^−17^ m^2^

**Table 2:** Parameters used for the calculation.

Sample	a (μm)	δ (μm)	n	p	r	E_0_ (MPa)	E_1_ (MPa)	ε_p_
1	41	40	20	1.1	1.04	61000	5000	0.0005
2	51	50	20	1.02	1.05	50000	5000	0.0005

## Data Availability

The data presented in this study are available on request from the corresponding author.
